# Analysis of four syringe dispensing machine point-of-access data 2017–2020 in Melbourne, Australia: machine utilisation and client demographics

**DOI:** 10.1186/s12954-022-00726-1

**Published:** 2022-12-21

**Authors:** Phoebe Kerr, Reece D. Cossar, Michael Livingston, David Jacka, Paul Dietze, Daniel O’Keefe

**Affiliations:** 1 Behaviours and Health Risks, Burnet Institute, 85 Commercial Road, Melbourne, VIC 3004 Australia; 2 Faculty of Health Sciences, National Drug Research Institute and enAble Institute, Curtin University, Perth, WA Australia; 3 Centre for Alcohol Policy Research, La Trobe University, Melbourne, VIC Australia; 4 Drug and Alcohol Service, Monash Health, Dandenong, VIC Australia; 5 School of Public Health and Preventive Medicine, Monash University, Melbourne, VIC Australia

**Keywords:** Syringe dispensing machine, Syringe vending machine, Needle and syringe program, Harm reduction

## Abstract

**Background:**

Australian needle and syringe distribution occurs via a mix of modalities, including syringe dispensing machines (SDMs). SDMs are electronic vending machines providing (often) 24-h access to needles/syringes and may attract greater numbers of people who are younger, female, and/or have limited connection to health care services compared to individuals accessing fixed-site needle and syringe programs (NSPs). However, validating the demographic characteristics of SDM clients has proven difficult in previous research.

**Methods:**

In this paper, we analyse SDM order and client demographic data from four SDMs located in South-East Melbourne, Australia, and compare this against the managing fixed-site NSP between May 2017 and December 2020. SDM data were collected via a novel 0–9 numeric keypad input tool. Via the tool, SDM clients were requested to input their categorised age, gender and postcode. Given the novelty of the tool, we evaluate the feasibility of the data collection method. We analysed data according to: (1) total SDM orders made, (2) estimated ‘unique SDM presentations’ and (3) describing the demographics of unique SDM clients. Importantly, we noted substantial invalid demographic data, and consequently, severely restricted data for analysis.

**Results:**

There were 180,989 SDM orders made across the four SDMs to an estimated 90,488 unique SDM presentations. There was little variation in unique presentations across days of the week, but 69% occurred out of NSP operating hours. Across the study period, the SDMs distributed 66% of the number of syringes distributed by the fixed-site NSP. Due to invalid demographic data, our restriction method provided only 10,914 (6% of all data) unique presentations for analysis. There were some demographic differences between SDM and NSP client, but these should be treated with caution.

**Conclusions:**

The data collection tool provides a novel means of comparing SDM and fixed-site presentations, demonstrating the substantial expansion of service via the SDMs. However, the validity of the demographic data was highly questionable and requires significant data coding, meaning it is not feasible for community NSPs. While we recommend the inclusion of automatically collected SDM order data, the use of a 0–9 numeric keypad to collect demographic data—while an innovation—requires alteration to support NSP data.

## Background

Needle and syringe (hereafter ‘syringe/s’) distribution in Australia increased from 40.3 million syringes per annum in 2011/12 to 50.2 million in 2020/21 [1]. The increase in syringe distribution is, in part, attributed to the expansion of needle and syringe program (NSP) services nationally, servicing an estimated population of approximately 74,000–93,000 people who inject drugs via 4218 outlets [1, 2]. Distribution of sterile injecting equipment occurs via a mix of primary (dedicated harm reduction services targeting people who inject drugs) and secondary (outlets ancillary to community or hospital health services) fixed-site NSPs, pharmacies, peer-to-peer distribution and outreach services [1]. Many NSPs also operate syringe dispensing machines (SDMs) to complement their fixed-site activities.

Despite being targeted to people who inject drugs, barriers to using fixed-site NSPs remain. These barriers may include fear of exposure by being recognised as a person who injects drugs, unwillingness to engage with NSP staff or inconvenient hours of operation [3, 4]. SDMs are electronic vending machines providing 24-h access to syringes and may help to alleviate some of the barriers associated with fixed-site NSPs. Some evidence suggests SDMs may also attract greater numbers of people who are younger, female, and/or have limited connection to health care services compared to individuals accessing primary NSPs or pharmacies [4–7]. There were an estimated 399 SDMs operating across Australia in 2021 [1], which potentially provide expanded harm reduction access to a more diverse range of people who inject drugs than fixed-site services, but findings around these client-type differences are inconsistent [8–13].

In research evaluating the effects of SDMs, different recruitment methods have been employed to recruit individuals who acquire syringes from SDMs, such as having researchers stationed adjacent to SDMs [9, 12], paper flyers advertising research located near SDMs [11] and within distributed SDM packs [9], internal timestamped counting devices [14] and viewing of CCTV footage at SDM sites [15]. All these methods have limitations and biases. For example, the times when researchers are stationed at SDMs may create bias by only capturing people who accessed the SDMs during stationed times and not those who might access the SDMs at other times (e.g., late at night). Similarly, paper survey forms or study advertisements are prone to self-selection bias, thereby potentially excluding people who are less likely to engage with services directly [9, 11].

To adequately capture the differences in clientele across various harm reduction modalities, and to better tailor services to meet client needs, novel research recruitment techniques are necessary. One such innovative system was used in Tbilisi, Georgia, by Otiashvili et al. [16] who employed an LCD screen attached to their SDMs. This interactive touch screen works in conjunction with individual plastic cards obtained by clients through fixed-site services. The cards track individual usage of the SDMs and the touch screen can be used both to purchase packs and to complete study surveys. The touch screens are able to provide general health messaging, instructional videos and access to optional client surveys. The survey covers basic data domains, such as socio-demographics and drug use history, and allows for baseline and six-monthly longitudinal surveying, as determined by the client card logons [17].

Timestamped transaction data were previously used by Duplessy and Reynaud [14] in an evaluation of Parisian SDMs, finding that most SDM presentations occurred during daylight hours—findings that contrast with other studies suggesting that SDM use often occurs outside fixed-site NSP operating hours [9, 11, 16]. A similar system was implemented in Melbourne, Australia, in 2017, using an innovative SDM data capture system. Four SDMs in the Greater Dandenong area of Melbourne employ not only automatic timestamp data but also require clients to enter basic, non-identifying demographic information (age category, gender and residential postcode) using a 0–9 keypad prior to ordering injecting equipment [18]. The introduction of such a system can provide insight into the characteristics of both SDM use and the demographics of SDM clients, thereby informing harm reduction activities. For example, we previously analysed trends in use of the four SDMs across times both during and not during COVID-19 city-wide lockdowns [18].

In this paper, we analyse the automatically collected SDM data to describe SDM presentations according to time/day variation in SDM presentations, and the client-entered demographic data to describe demographic make-up of SDM clients. We compare these data against the presentation data of the fixed-site NSP that manages our study SDMs. Given the novelty of the data collection methods, we also aim to evaluate the feasibility of collecting demographic and timestamped data via this system. We aim to understand whether self-reported demographic data using the numbered keypad system provide an accurate understanding of who utilises the SDMs. Collecting data via the keypads may offer a simple and novel method of data collection, potentially overcoming some of the above limitations to SDM research.

## Methods

### Syringe dispensing machines

**Fig. 1:**
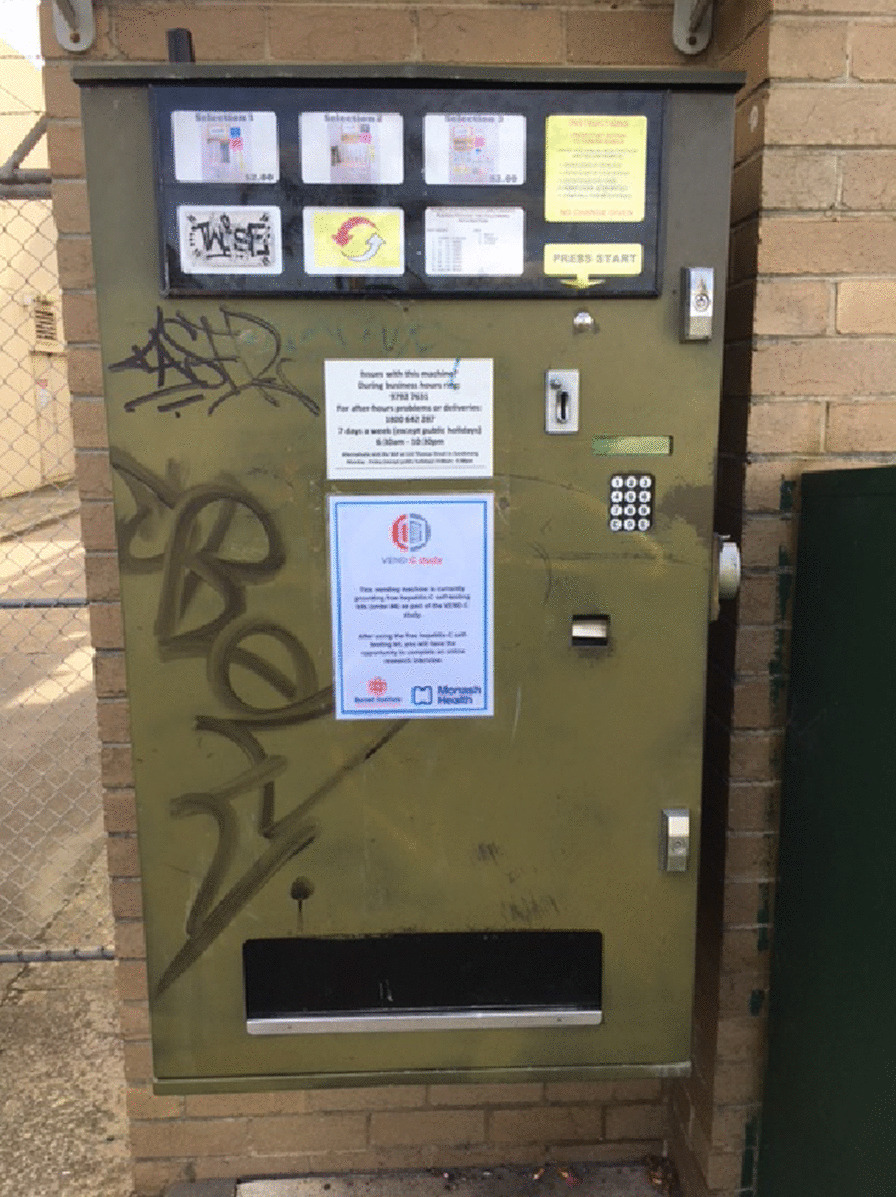
Image of the Dandenong SDM

The SDMs are at four locations across south-eastern Melbourne (Dandenong, Casey, Pakenham and Monash) [18] and are all separate from the fixed-site NSP that manages the four SDMs (Monash Community NSP, MCNSP). The SDMs are relatively discreet units, positioned in obscured locations, with non-conspicuous signage and design (see Fig. 1) and are available for use 24 h a day, seven days a week. The SDMs all distribute three different packs of injecting equipment: a ‘free pack’ (eight 1 ml syringes, two needle stick disposal units); a AUD$2 ‘water pack’ (six 1 ml syringes, two sterile water ampoules and two needle stick disposal units); and a AUD$2 ‘pill filter pack’ (one sterile water ampoule, one pill wheel filter, one 3 ml syringe barrel and three separate attachable needles (19, 21 and 25 gauge). All packs include two alcohol swabs, one plastic spoon, one cotton wool filter, one condom and water-based lubricant and one health promotion leaflet (Table 1). From May 2017 to August 2017, the ‘free pack’ contained only six 1 ml syringes.

**Table 1:** SDM pack contents (as of September 2017)

Free pack	Water pack ($2AUD)	Pill filter pack ($2AUD)
Eight 1 ml syringes Two syringe disposal units Two alcohol swabs One cotton wool filter One plastic spoon One condom One lubricant sachet One health promotion leaflet	Six 1 ml syringes Two sterile water ampoules Two syringe disposal units Two alcohol swabs One cotton wool filter One plastic spoon One condom One lubricant sachet One health promotion leaflet	One 3 ml syringe barrel Three attachable needles (19, 21, 25 gauge) One sterile water ampoule One pill wheel filter Two alcohol swabs One cotton wool filter One plastic spoon One condom One lubricant sachet One health promotion leaflet

Following injection, clients may dispose of used needles/syringes at multiple, accessible locations, including inside fixed-site NSPs, and in 24-h accessible disposal bins outside fixed-site NSPs.

### Syringe dispensing machine data capture

**Fig. 2:**
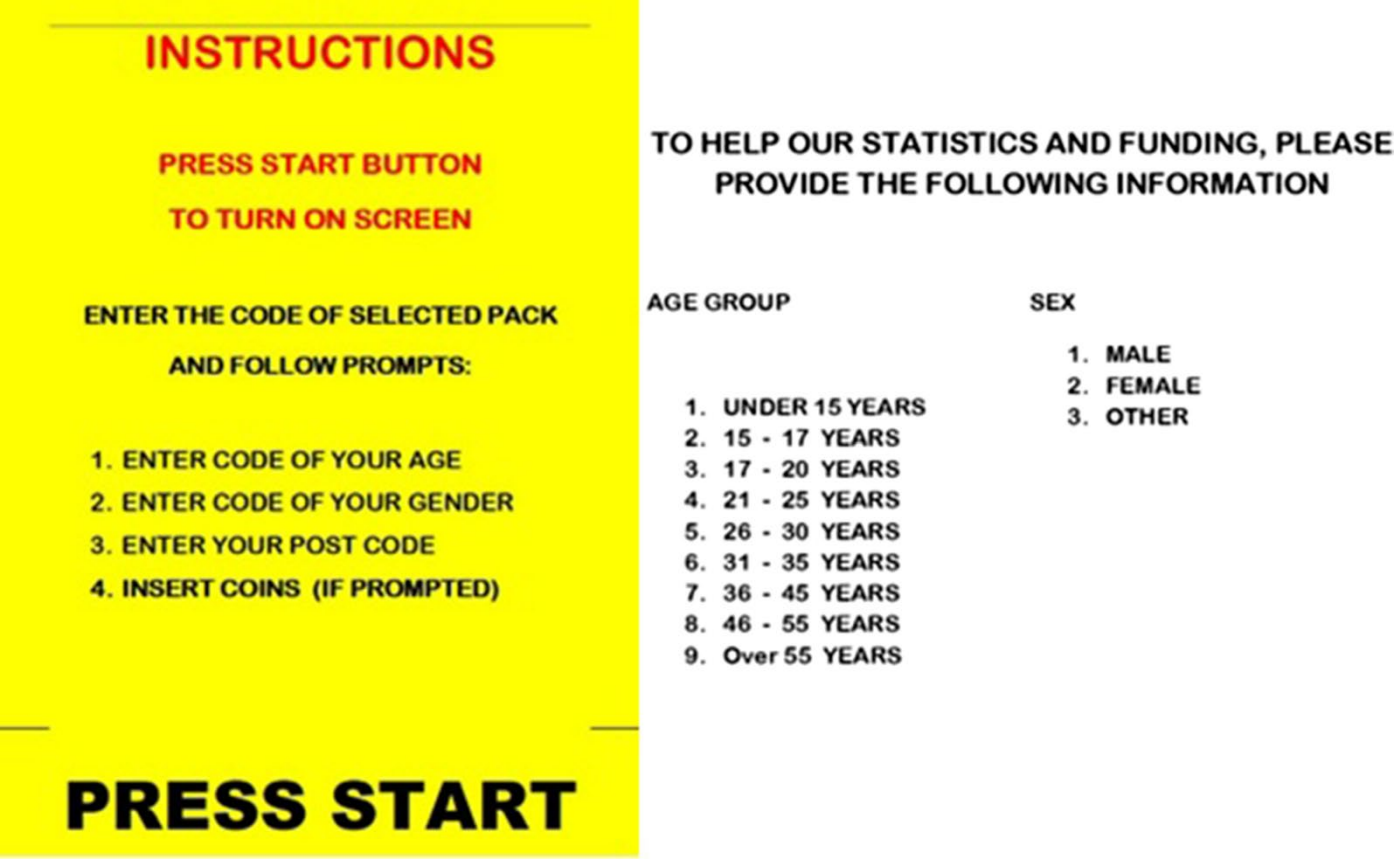
SDM demographic data entry instructions

The SDMs automatically capture data on the time/day and order type/cost (including failed orders or machine malfunctions) for each order placed. Prior to dispensation of syringe orders, the SDM requires clients to enter basic demographic data using a 0–9 numeric keypad, entering their information according to pre-determined categories: age category (1 < 15 years, 2 = 15–17 years, 3 = 18–20 years, 4 = 21–25 years, 5 = 26–30 years, 6 = 31–35 years, 7 = 36–45 years, 8 = 46–55 years and 9 > 55 years), gender (1 = male, 2 = female, 3 = ‘other’) and residential postcode (Fig. 2). Age categories match those entered for clients accessing fixed-site NSPs. We obtained SDM data from 1 May 2017 to 31 December 2020. Due to machine error, data were missing for the Casey and Dandenong SDMs during August 2017 (in August of other years, the Casey SDM had between 316 and 479 presentations, and the Dandenong SDM between 2590 and 3020 presentations). A further 134 records were lost from the Pakenham SDM in December 2018, again due to machine error.

### Fixed-site NSP reporting data

Victorian NSPs are required to collect and report on basic service client demographic data via the Needle and Syringe Program Information System (NSPIS). Client categorised age and gender were provided by the MCNSP. The number of syringes dispensed by the MCNSP are reported also. For two reporting months (February and April 2019), reported syringe dispensation was much higher than other months. This was confirmed as an error by the MCNSP. For these two months, data were imputed by replacing the erroneous figures with the average syringe dispensation across all other months.

### Data analysis

We previously published analysis of trends in SDM use across the same time period for the four SDMs [18], and given the proposed scope of this paper, we limit analysis of data to describing presentations and basic SDM client demographics using the three methods described below:

1. *Descriptive analysis of total SDM orders:* we report on access and syringe dispensation across total orders made via the four SDMs. In this instance, we refer to ‘orders’ as the dispensation of a single pack of injecting equipment, even if a person then made multiple orders in a single presentation (e.g. a person orders 10 injecting packs in a single presentation = 10 ‘orders’).
2. *Descriptive analysis of estimated unique SDM presentations:* we refer to ‘unique presentations’ as SDM access by unique individuals or groups of individuals (irrespective of how many SDM orders were placed during a single unique presentation; e.g. a person orders 10 injecting packs in a single ‘unique presentation’ = one ‘unique presentation’). To clarify, we can only attempt to estimate the uniqueness of presentations (even if made by the same person), rather than estimate the total number of unique SDM clients, given there is no way to separately identify individuals accessing the SDMs (such as by using a registration number).
  To estimate SDM presentations, we applied a 45-s cut-off between SDM orders. We assumed that a person could make any one SDM order within 45 s of each other, and that any multiple orders occurring within 45 s of one another were part of a single unique presentation. Therefore, any order occurring more than 45 s since the previous order was assumed to be a different unique presentation by another person. We report access and syringe dispensation across unique presentations. Further, we analysed the time/day of unique presentations, comparing this against the opening hours of the MCNSP, which operates 9am–5pm, Monday to Friday.
3. *Descriptive analysis of SDM client demographic data:* Preliminary data inspection indicated many SDM demographic data entries were invalid. For example, 44,195 SDM orders (24% of total 180,989 orders) inputted data for gender, age category and postcode that were simple repetitions of the same data entry keypad number (e.g., gender = 1, age category = 1 and postcode = 1111), thereby indicating invalid demographic data (not dispensation data, which are automatically collected by the SDMs). According to other criteria (outlined below), we conservatively estimated that approximately 50% of all SDM orders inputted invalid demographic data. We therefore sought to restrict our analyses to include only the most potentially valid demographic data.
  First, we compared postcode data against a list of Australian postcodes [19] and removed all records without a valid Australian postcode (*n* = 16,462, 9% of all order data, including 5061 instances where postcode data were missing due to the SDM being sold out or otherwise some other machine malfunction in recording postcode data). We then removed all postcodes that were not within the state of Victoria (where the four SDMs are located) (*n* = 51,391, 28% of all order data). We then removed all postcodes that were ‘3333’, assuming this to be repetitive inputting; even though ‘3333’ is a legitimate Victorian postcode, it is the postcode of Meredith, a town very far from where the SDMs are located and with a small population (*n* = 3609, 2% of all order data). Then, we removed all instances where a client inputted the same number for product type, gender and age category (i.e. product=1, gender = 1 and age category = 1) (n=17,054, 9% of all order data). This initial data cleaning process removed 88,516 (49% of all order data) records from the dataset.
  Second, after removing what we determined as invalid demographic data, we identified clients with more than one SDM order within a single unique presentation using our 45-s cut-off (meaning a client came to the SDM and made multiple orders). Using demographic data from the first order, we matched age category, gender and postcode data against the age category, gender and postcode data from the next subsequent SDM order. By this methodology, we attempted to validate a client’s demographic data against the same information obtained within the 45-s window. This validation method provided 10,914 (6% of all order data) unique presentation records for analysis﻿.

Comparative MCNSP data were only available as aggregated monthly data, with data available from May 2017 through to December 2020 (*n* = 44 months).

## Results

### Descriptive analysis of total SDM orders

There were 180,989 SDM orders made across the four SDMs (Casey = 16,922 (9.35%); Dandenong = 113,341 (62.62%); Monash = 29,928 (16.54%); and Pakenham = 20,798 (11.49%)) during the analysis period. Across total SDM orders, 175,551 (97.00%) successfully distributed injecting equipment, with 559 (0.30%) instances of machine malfunction and 4879 (2.70%) instances where the selected injecting equipment was sold out/unavailable.

SDM order data were still captured even if nothing was dispensed (due to a machine jam, for example). In total, there were 168,183 (92.92%) orders of a free pack, 8448 (4.67%) orders of a water pack and 4358 (2.41%) orders of a pill filter pack. There were minor differences in product orders among successfully dispensed orders; however, the water pack and pill filter pack were listed as ‘sold out’ during 11.88% and 9.11% of their orders (compared to 2.07% of free pack orders), suggesting a potential underestimate in demand for these products. A total of 1,347,073 syringes were provided via all successful SDM orders. The MCNSP reported distributing a total of 2,037,411 syringes during the analysis period.

### Descriptive analysis of estimated unique SDM presentations

We estimated there were 90,488 unique presentations to the SDMs. The distribution of unique presentations across the four SDMs was relatively consistent with total orders; Casey = 9513 (10.52%); Dandenong = 56,731 (62.69%); Monash = 15,066 (16.65%); and Pakenham = 9178 (10.14%). The median number of SDM orders during unique presentations was 1 (IQR: 1–2, range 1–120; the 120 attempts due to the SDM being sold out of product). The median number of needles/syringes successfully dispensed during unique presentations was 8 (IQR: 8–16, range: 1–552).

**Fig. 3:**
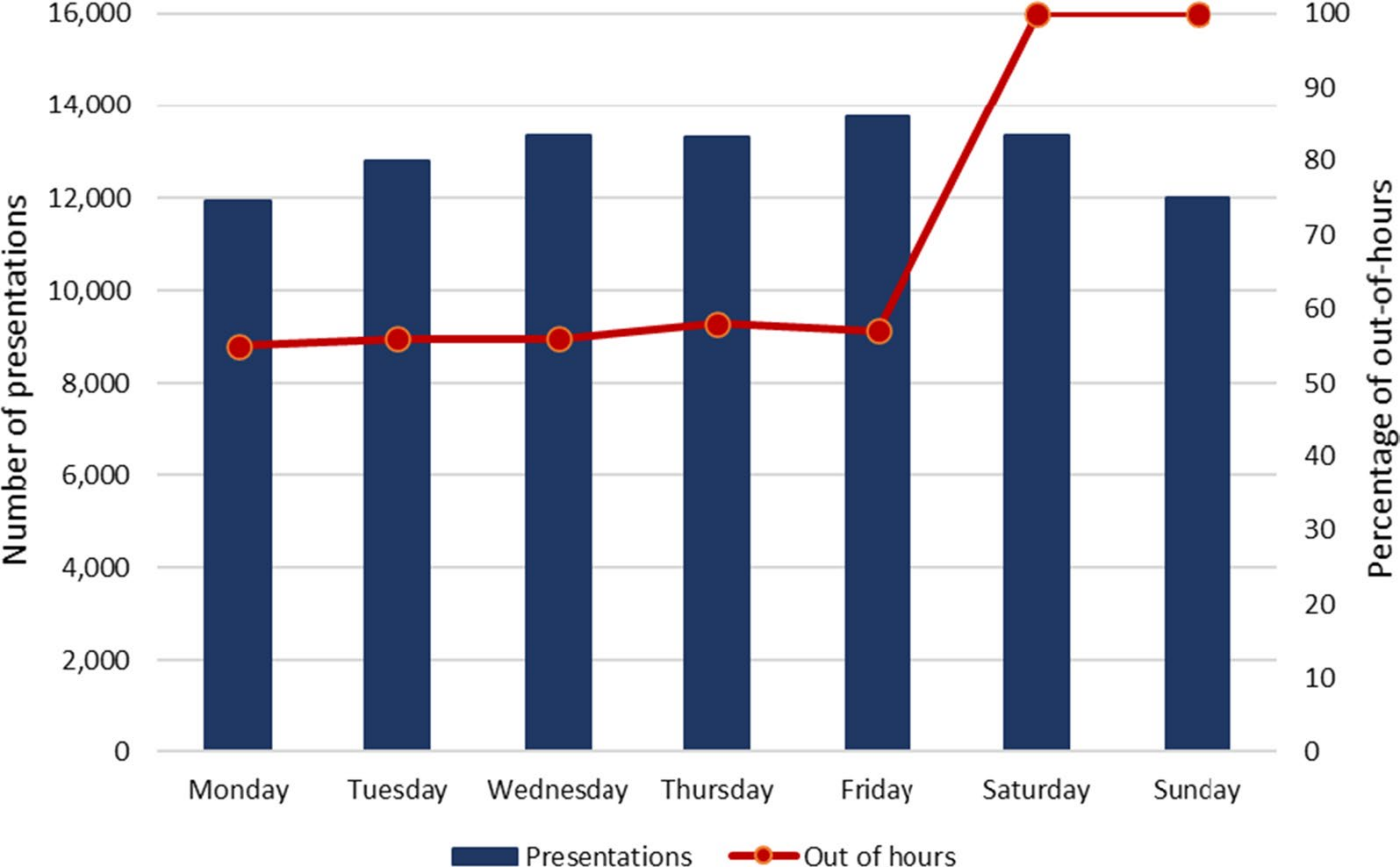
Unique SDM presentations occurring across the week and during NSP operating hours

There was little variation in unique presentations across days of the week, with total presentations ranging between 11,946 (13.20% of all unique presentations) and 13,742 (15.19%) across all days. Sixty-nine per cent (*n* = 62,093) of all unique presentations occurred out of NSP operating hours, again with little variation across weekdays, with between 54.94% and 57.86% of presentations occurring out of hours from Monday to Friday. (100% of presentations on Saturday and Sunday were categorised as occurring out of hours, when the MCNSP was closed, as shown in Fig. 3.)

Over the study period, the MCNSP reported 33,922 unique presentations, or approximately 37.48% of estimated unique SDM presentations.

### Descriptive analysis of SDM client demographic data

We report here only on data from the 10,914 unique presentations that conformed to our definition of valid demographic data (12% of all unique presentations). Among these presentations, 54.23% (*n* = 5919) reported male gender, 39.89% (*n* = 4354) reported female gender, and 5.88% (*n* = 641) reported a non-binary (‘Other’) gender (Table 2). The majority of presentations reported an age > 30 years (*n* = 6783, 62.15%); however, 13.33% of presentations (*n* = 1455) reported an age ≤ 20 years. This was particularly noted among presentations reporting a non-binary gender, among whom 60.53% (*n* = 388) reported an age ≤ 20 years.

**Table 2:** Reported age category and gender among clients with repeated SDM orders during a single ‘unique’ presentation, *n*(%)

Age category	Male gender	Female gender	‘Other’ gender	Total
< 15 years	218 (3.7)	57 (1.3)	61 (9.5)	336 (3.1)
15–17 years	137 (2.3)	16 (0.4)	54 (8.4)	207 (1.9)
18–20 years	182 (3.1)	457 (10.5)	273 (42.6)	912 (8.4)
21–25 years	565 (9.5)	232 (5.3)	14 (2.2)	811 (7.4)
26–30 years	1019 (17.2)	793 (18.2)	53 (8.3)	1865 (17.1)
31–35 years	891 (15.1)	679 (15.6)	58 (9.0)	1628 (14.9)
36–45 years	2036 (34.4)	1273 (29.3)	45 (7.0)	3354 (30.7)
46–55 years	629 (10.6)	719 (16.5)	23 (3.6)	1371 (12.6)
> 55 years	242 (4.1)	128 (2.9)	60 (9.4)	430 (3.9)
Total	5919 (100)	4354 (100)	641 (100)	10,914 (100)

Some differences in SDM access were noted, with SDM clients ≤ 25 years more often accessing the SDM out of hours compared to SDM clients > 25 years (72% vs. 67%, *p* value ≤ 0.001). Gender differences were also noted, with males more often accessing the SDMs out of hours compared to females and the ‘other’ gender category (69% vs. 68% vs. 64%, respectively, *p* value = 0.008).

Over the analysis period, the MCNSP reported age category and gender data for 33,412 presentations (98.49% of total MCNSP presentations). From these, 79.02% (*n* = 26,405) reported male gender and 20.95% (*n* = 7001) reported female gender. (Six presentations did not report gender data.) Presentations reporting an age > 30 years accounted for 87.67% (*n* = 29,294) of all fixed-site presentations, while presentations reporting an age ≤ 20 years accounted for only 0.5% (*n* = 176) of all presentations.

## Discussion

### SDM dispensation data

Our automatically collected point-of-access data tool provides a novel means of comparing SDM presentations with on-site presentations to a primary fixed-site NSP with results demonstrating the substantial expansion of fixed-site NSP service via the SDMs. Across the study period, the SDMs distributed 66% of the number of syringes distributed by the fixed-site NSP, despite SDM dispensation restrictions (e.g., limited number of syringes per order).

Previous research has reported inconsistent findings related to times of SDM use [9, 11, 14]. Our findings contrast to those of the 12-year evaluation of a Parisian SDM network by Duplessy and Reynaud [14]. The majority of unique presentations in our analysis occurred outside of the MCNSP operating hours, consistent with previous Australian research [9, 11]. As the MCNSP is closed on weekends, the SDMs represented an alternative NSP modality for these times, providing for client needs [4, 20], particularly for certain sub-groups of SDM clients. However, one of the disadvantages of SDMs is the lack of contact with NSP staff, thereby potentially reducing opportunities for health referrals and treatment pathways [4, 20], and while there is potential for SDMs to be out-of-stock or malfunctioning [9, 14], we found limited evidence of this (~ 3% of all orders). Another disadvantage is product capacity and resources needed to fill the SDM when products become unavailable (e.g., during the weekends). In response, the MCNSP previously increased the number of syringes included in the dispensed free packs from six to eight, enabling SDM clients to acquire more syringes in a single SDM visit. This change was implemented following feedback from an informal survey conducted by the MCNSP staff. Despite these potential limitations, the SDMs clearly offer a service acceptable to many individuals as a convenient and accessible intervention, providing sterile injecting equipment during times when the fixed-site NSP is otherwise closed, such as during work/school hours.

### SDM client demographic data and evaluation of method

While the SDMs automatically recorded valid data on the time, day and type of product ordered, demographic data were manually entered by SDM clients. In attempting to estimate the demographic make-up of unique SDM presentations, compared to the MCNSP, we limited data to what appeared to be valid estimates of client characteristics. As an evaluation of the demographic data input tools used by the analysed SDMs, we recognise that they are an innovation, however, our analysis highlights major limitations with their use. We estimated that over half of all demographic data were inputted invalidly, most commonly by the client deliberately inputting incorrect data (often by simply repeating the same numeric input). Our methods of attempting to identify valid data led to further reductions, meaning we only analysed 6% of all available data. Further, we cannot be sure that the individuals who entered what we classified as valid data were the same as those who did not (deliberately or otherwise) enter valid data. For these reasons, and without external validation of our assumptions, our findings below need to be treated with caution.

Australian studies and national reports describing the age and gender of people who inject drugs who access NSPs have consistently reported majority proportions of males aged 30 years or older [12, 21, 22]. In the 2021 National Data Report from the Australian NSP Survey, only 2% of people attending NSPs were aged < 25 years [22]. Sixty-three per cent of people were male, 35% were female, and 1% reported their gender as ‘other’ [22]. This was broadly comparable to client presentation data from the MCNSP. Our SDM client demographic estimates, if accurate, suggested higher percentages of women, non-binary genders and young people compared to the MCNSP. While questionable in terms of validity, these findings do correspond to previous international outcomes [4–6] and may reflect the barriers in access to health services for individuals with a different profile from the dominant identity. While SDMs allow for access to sterile injecting equipment by not accessing fixed-site NSPs, these populations may be missing opportunities for healthcare referrals and treatment pathways that fixed-site NSPs facilitate [17, 23]. This is particularly pertinent as primary fixed-site NSPs (such as the MCNSP) serve as an important point of access for wider healthcare provision [23, 24]. Previous research has stressed the need to reduce barriers to fixed-site NSPs so that they can be more responsive to a greater diversity of clients, such as women, young people and LGBTQ+ people [25–29]. Primary fixed-site NSPs and other distribution modalities become more effective in servicing their clientele by understanding and responding to the social, organisational and political context in which they are situated [6, 24].

While we treated data using a method that we believe approached validity, there remains distinct uncertainty in our methods, and believe that such analysis is inappropriate for NSPs seeking to monitor service delivery. Automatically collected SDM order data are inherently valid (so long as the SDM is appropriately functioning) and should be included within all SDMs. However, our evaluation of the SDM data suggests the numeric keypad is not a feasible method of collecting client demographic data. Consequently, alternative approaches and solutions are recommended when using similar data collection methods. It may be that in some cases, invalidly entered demographic data were not an attempt by the SDM client to obscure personal information, but impatience or frustration with the data input process (particularly when making multiple orders). As an initial mitigation measure, the keypad numbers assigned to demographic variables could be different from those assigned to order the various packs of injecting equipment (potentially dissuading clients to simply enter the same number for all inputs). Alternatively, the touch screen keypad as utilised by Otiashvili et al. [16] allows for specific questions and answers to be presented to clients, thereby enabling the inclusion of questions specifically asking clients to choose between ‘male’/‘female’/‘non-binary’, rather than response designations to a numeric keypad. Although not all SDM systems may have the resources to develop and install such technology, this could be an additional solution to data quality issues. Finally, other questions exploring client ethnicity or more detailed gender diversity (rather than a basic ‘other’ category) are recommended to aid in further understanding the needs of different subpopulations of people who inject drugs, although any additional questions need to be balanced against the convenience of accessing SDMs.

## Limitations

There are several limitations to our study. Most important is the issue of demographic data validity, the limitations of which are described above. Further, while we assumed that 45 s was a sufficient cut-off to distinguish unique individuals making SDM presentations, there is no way to validate this and we almost certainly included multiple individuals within the same ‘unique’ presentation, particularly if two individuals came to the SDM together. Therefore, we cannot know how many unique people who inject drugs account for our estimate of SDM presentations, nor if some people who inject drugs access syringe distribution services other than the SDM (such as the NSP). However, we repeat that an aim of this paper was to evaluate the feasibility of the data collection method, and these are important lessons to be learned.

There were other limitations with our data, including missing data due to machine malfunction. This malfunction impacted only some of the SDMs and only for a very temporary period, representing a fraction of total SDM data.

Finally, because we could not uniquely identify individuals, we could not segregate their data, meaning unique individuals were undoubtably counted multiple times in the demographic data. This could impact the data by a particularly frequent SDM user disproportionately contributing to data, or an individual contributing to multiple categories, should they change age or gender categories within the study period. However, the latter is unavoidable, and due to the relatively short study period (approximately three and a half years), anticipated to occur very infrequently. Further, this is also a limitation of existing NSP data, against which the SDM data were compared.

## Conclusion

Our analysis explored a novel method of collecting SDM data to evaluate both SDM use and the demographics of clients who utilise SDMs in comparison with those who frequent fixed-site NSPs. While automatically collected SDM order data are a useful tool for demonstrating the substantial value add of SDMs to expand NSP service delivery, further refinement and innovation is needed for reliable demographic data collection. The numeric keypad tool described here is certainly an important, initial development in SDM data collection, which has traditionally posed intractable issues for public health research. Our analysis seemed to support previous suggestions about the differences in SDM clients, but due to the limitations of the data collected, we cannot be certain of our results. However, through suggested modifications, such as touch screen technology, the SDM data collection could prove a powerful tool in supporting international harm reduction services to tailor their intervention for a broader and more diverse clientele.

## Data Availability

The datasets generated and/or analysed during the current study are not publicly available but may be available from the data custodian (david.jacka@monashhealth.org) on reasonable request.
